# Disparities in Video and Telephone Visits Among Older Adults During the COVID-19 Pandemic: Cross-Sectional Analysis

**DOI:** 10.2196/23176

**Published:** 2020-11-10

**Authors:** Christopher H Schifeling, Prajakta Shanbhag, Angene Johnson, Riannon C Atwater, Claire Koljack, Bennett L Parnes, Maria M Vejar, Samantha A Farro, Phoutdavone Phimphasone-Brady, Hillary D Lum

**Affiliations:** 1 Division of Geriatric Medicine University of Colorado School of Medicine Aurora, CO United States; 2 Department of Psychiatry University of Colorado School of Medicine Aurora, CO United States; 3 VA Eastern Colorado Geriatric Research Education and Clinical Center Aurora, CO United States

**Keywords:** telemedicine, telehealth, telephone, videoconferencing, health care disparities, older adults, geriatrics, advance care planning, advanced directives, COVID-19, coronavirus pandemic, SARS-CoV-2, primary care

## Abstract

**Background:**

Telephone and video telemedicine appointments have been a crucial service delivery method during the COVID-19 pandemic for maintaining access to health care without increasing the risk of exposure. Although studies conducted prior to the pandemic have suggested that telemedicine is an acceptable format for older adults, there is a paucity of data on the practical implementation of telemedicine visits. Due to prior lack of reimbursement for telemedicine visits involving nonrural patients, no studies have compared telephone visits to video visits in geriatric primary care.

**Objective:**

This study aimed to determine (1) whether video visits had longer durations, more visit diagnoses, and more advance care planning discussions than telephone visits during the rapid implementation of telemedicine in the COVID-19 pandemic, and (2) whether disparities in visit type existed based on patient characteristics.

**Methods:**

We conducted a retrospective, cross-sectional analysis of patients seen at two geriatric clinics from April 23 to May 22, 2020. Approximately 25% of patients who had telephone and video appointments during this time underwent chart review. We analyzed patient characteristics, visit characteristics, duration of visits, number of visit diagnoses, and the presence of advance care planning discussion in clinical documentation.

**Results:**

Of the 190 appointments reviewed, 47.4% (n=90) were video visits. Compared to telephone appointments, videoconferencing was, on average, 7 minutes longer (mean 37.3 minutes, SD 10 minutes; *P*<.001) and had, on average, 1.2 more visit diagnoses (mean 5.7, SD 3; *P*=.001). Video and telephone visits had similar rates of advance care planning. Furthermore, hearing, vision, and cognitive impairment did not result in different rates of video or telephone appointments. Non-White patients, patients who needed interpreter services, and patients who received Medicaid were less likely to have video visits than White patients, patients who did not need an interpreter, and patients who did not receive Medicaid, respectively (*P*=.003, *P*=.01, *P*<.001, respectively).

**Conclusions:**

Although clinicians spent more time on video visits than telephone visits, more than half of this study’s older patients did not use video visits, especially if they were from racial or ethnic minority backgrounds or Medicaid beneficiaries. This potential health care disparity merits greater attention.

## Introduction

The COVID-19 pandemic presents multiple challenges for older adults with regard to medical care access. This population is at the highest risk for morbidity and mortality from coronavirus infection [1]. Furthermore, physical distancing efforts to reduce this risk have disrupted medical care for more than half of all adults over 70 years of age [2]. These interruptions are particularly problematic, given the high prevalence of multimorbidity and frailty in this population [3]. More than 1 in 7 older adults have experienced a disruption in what they considered essential medical services due to the pandemic [2].

One approach to maintaining access to care while reducing the risk of community spread is telemedicine appointments [4]. Telemedicine, which is sometimes used interchangeably with telehealth, refers to using electronic information and communication technologies to provide health care when distance is a barrier [5]. These telecommunication systems can range from messages through electronic patient portals to live, synchronous interactions through audio and video communication [6]. Although telemedicine services have been used prior to the COVID-19 pandemic, they were not reimbursed at the same rate as in-person visits [7]. In response to the rapid expansion and use of telemedicine services during the rise of the pandemic, the Centers for Medicare and Medicaid Services expanded the scope and rate of telemedicine reimbursement, including equivalent reimbursement for telephone and video visits to match payments for similar outpatient visits [6].

Systematic reviews of telemedicine use in older adult populations suggest that older adults generally accept, and are satisfied with, the use of telemedicine [8-10]. However, most telemedicine studies with this population have had a small number of participants, have been performed within the Department of Veterans Affairs, or have focused on specific problems (ie, dementia) rather than the management of multimorbid patients [9-15]. The types of telemedicine services (ie, video visits and phone visits) that can meet the needs of geriatric patients in clinical practice has not been evaluated [16].

The ability of older patients, family care partners, and health care providers to discuss common medical issues, such as management of multiple chronic conditions and advance care planning via either type of telemedicine visit, is unknown. Addressing disease-related vulnerabilities and advance care planning is particularly important in the setting of the COVID-19 pandemic, as the risk of critical illness has become more imminent [17]. However, advance care planning and other conversations about serious illness can be difficult for patients and their families, as they often require significant time and attention to emotional cues and may be limited by telemedicine visits.

Although video-based visits, compared to telephone visits, may improve communication through the addition of facial cues, they may be especially difficult for older adults because they require specific equipment, internet access, and technology navigation [10]. The increased prevalence of impaired cognition, hearing, vision, and dexterity in this population also poses particular problems [10,16]. However, the expanded reimbursement policy for telemedicine significantly favors video visits (ie, two-way, synchronous communication with audio and video) over audio-only communication (ie, telephone visits) [6].

The principal aim of this study was to describe the rapid transition to telemedicine (ie, telephone and video visits) to meet the needs of geriatric primary care patients during the COVID-19 pandemic. We hypothesized that video visits might have more capacity to address multimorbid disease (as indicated by visit duration and number of visit diagnoses) and advance care planning discussions. We also explored whether there were health disparities between video visits and telephone visits based on patient sociodemographic factors.

## Methods

### Design and Setting

In this retrospective, cross-sectional study, we performed an electronic health record (EHR) review of charts used in video and telephone appointments at 2 primary care clinics in Colorado for patients aged 75 years or older (ie, enrollment age for the clinics). Neither clinic offered telemedicine appointments prior to the COVID-19 pandemic. One clinic is located directly at a large academic medical center with 13 geriatric medicine clinicians, 4 geriatric fellows, and 1 psychologist working a total of 6 full-time equivalents. The other clinic is located at a free-standing outpatient center with 3 geriatric medicine clinicians and 1 psychologist working a total of 1 full-time equivalent. Both clinics use the same EHRs and have the same capacity for telemedicine visits. Patients who requested a routine or acute care visit were offered a choice between video and telephone-based visits. This evaluation was conducted as part of the Comprehensive Primary Care Plus’ quality improvement activities, and institutional review board approval was not required.

### Participants

In total, approximately 25% of the patients who had telephone and video appointments with the clinics from April 23 to May 22, 2020 were included in this study. Participant selection was performed by first identifying all patient encounters and then using a computerized randomization process to select patients who would go through manual chart review by 4 authors. In Colorado, in-person clinic visits stopped on March 16, 2020, due to the community spread of COVID-19. The starting date for chart review was 6 weeks after both clinics converted to conducting telemedicine visits. This start date allowed for data collection from appointments that occurred after addressing the initial challenges of rapidly implementing video and telephone visits. In order to ensure that the analysis only included unique patients, only 1 visit encounter was allowed per patient. Although the number of patients who visited multiple times during the study period is unknown, none of the randomly selected visits for chart review were from repeat visits.

### Data Collection

We extracted patient and visit characteristics from EHRs using a standardized data collection tool. Study data were managed using the REDCap (Research Electronic Data Capture; Vanderbilt University) electronic data capture tools at the University of Colorado [18]. Patient characteristics included age, gender, race, ethnicity, insurance status, need for an interpreter, patient portal account status (ie, active or inactive), and specific impairments that might affect telemedicine (ie, cognitive, auditory, and visual impairment), which were determined based on the presence or absence of the impairment in our ICD-10 (International Statistical Classification of Diseases, 10th Revision)–based problem list. Cognitive impairment was determined by diagnoses that involved dementia, memory loss, or cognitive impairment. Auditory impairment was determined by diagnoses that involved hearing aids or hearing impairment. Visual impairment was determined by the diagnoses listed in the “Eye” section of patients’ active problem list, and included a wide range of diagnoses common among older adults.

Visit characteristics, which were obtained from the prompts in the note template of EHRs, included type of visit (ie, telephone or video), provider type (ie, behavioral health or geriatric medicine), type of video system used (eg, health system’s patient portal video function or other video systems, such as the Doximity Video [Doximity Inc]), and reasons for why a video system was not used for the visit, if applicable. An acute visit was determined by the presence of specific symptoms in the “reason for visit” section, which was documented by medical assistants during phone calls with patients conducted prior to appointments. Clinician notes were reviewed for documentation on vital signs, which were measured at patients’ homes, and the presence of a care partner during the visit. For video visits, documentation from the physical exam was reviewed to determine whether there were findings that relied on visual observation and could not have been assessed with audio-only communication. These visual observation-dependent findings could either be visual observations that were noted outside of the constitutional section or descriptions of patients’ home environment. The number of medication changes was determined from documentation automatically created by the EHR based on clinicians’ orders during the encounter, which includes medications that are started, changed, or stopped. Data on whether patients viewed the after-visit summary through the patient portal were recorded automatically by the EHR in the encounter documentation.

The outcomes (ie, visit duration, number of visit diagnoses, and presence of advance care planning discussions) were obtained from clinical documentation. Visit duration was based on clinician documentation and was in line with reimbursement requirements at the time of this study. Specifically, the time documented reflected the time spent with the patient and associated counseling or coordination of care. This study was completed prior to the new documentation guidance, which stated that telehealth visits could include reimbursement for the provider’s total time spent, including preparation, visit, and postvisit times on the same day. The number of visit diagnoses was based on clinicians’ ICD-10 diagnoses during the encounter. The visit note template used by both clinics prompted clinicians to discuss advance care planning (eg, discussions on choice of medical power of attorney and code status preferences). These types of advance care planning discussions reflected documentation that was present in the specific visit, but did not reflect whether a patient had prior advance care planning discussions or documentation in the EHR or at home.

### Statistical Analysis

We compared descriptive statistics for patient and visit characteristics between telephone visits and video visits, using *t* tests for continuous variables and Chi-square tests for categorical variables. Continuous and categorical variables were expressed as means with standard deviations and percentages, respectively. Multivariable linear and logistic regression models were used to evaluate the relationship between visit type (ie, the independent variable) and the following 4 dependent variables: visit duration, number of diagnoses, discussion of medical durable power of attorney, and code status discussion. These models were adjusted for the following covariates: age, need for an interpreter, Medicaid beneficiary status, and presence of a care partner. All tests for statistical significance were two-tailed, and a *P* value of <.05 was considered statistically significant. All statistical analyses were done using SAS version 9.4 (SAS Institute).

## Results

**Figure 1:**
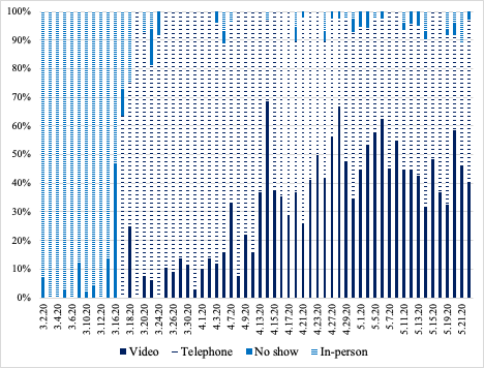
Stacked histogram showing the percentage of visit types over time from March through May 2020. There were no video or telephone visits prior to March 17, 2020 when the clinic initially began to implement telemedicine appointments in response to the COVID-19 pandemic. For the first several weeks after implementing telemedicine appointments, the majority of visits were done by telephone. Video visits rose in prevalence throughout April.

In a 6-week period during the COVID-19 pandemic, during which almost no in-person clinic visits were possible, the clinics had a combined total of 424 scheduled telephone visits and 384 scheduled video visits (Figure 1). After March 18, 2020, the daily no-show rate ranged from 0% to 14%, which was similar to the pre-COVID-19 no-show rate. This overall volume of visits represents 85% of visits prior to the COVID-19 pandemic. Of the 25% of visits randomly selected for inclusion in this study, 9 appointments were excluded from chart review because 7 patients did not arrive for their appointments and 2 appointments were only brief follow-up phone calls for recent appointments, not full appointments. In total, 190 appointments underwent chart review, including 100 telephone visits and 90 video visits.

Of the 190 appointments (Table 1), 70% (n=133) of patients were female, 15.8% (n=30) were Black, 13.2% (n=25) needed interpreters, and 18.9% (n=36) had Medicaid coverage. The average age was 82.5 years (SD 6.2 years). There was a high prevalence of hearing, vision, and cognitive impairment. Caregivers were present for 25.3% (n=48) of appointments. The number of caregivers present prior to the COVID-19 pandemic is unknown. Most patients (137/190, 72.1%) had active electronic patient portals, regardless of whether they had a telephone or video visit. Patients who had video visits were younger (mean 81.3 years, SD 6.4 years vs mean 83.5 years SD 5.9 years; *P*=.01), more likely to have an active patient portal account (n=81, 59.1% vs n=56, 40.9%; *P*<.001), and more likely to have a caregiver present during the visit (n=31, 64.6% vs n=17, 35.4%; *P*=.01) compared to patients who had telephone visits. Non-White patients, patients who needed an interpreter, and Medicaid beneficiaries, were less likely to have video visits than White patients, patients who did not need an interpreter, and non-Medicaid beneficiaries (*P*=.003, *P*=.01, *P*<.001, respectively). There were no differences in the likelihood of video visits based on cognitive, auditory, or visual impairments.

**Table 1:** Patient and visit characteristics of appointments reviewed listed by visit type (N=190).

Patient and visit characteristics		All visits		Telephone visit^a^ (n=100)		Video visit^a^ (n=90)		*P* value	
Age, years (mean, SD)		82.5 (6.2)		83.5 (5.9)		81.3 (6.4)		.01	
Sex - Female, n (%)		133 (70)		68 (51.1)		65 (49.9)		.53	
**Race, n (%)**								.003^b^	
	Asian		18 (9.5)		12 (66.7)		6 (33.3)		N/A^c^
	Black		30 (15.8)		24 (80)		6 (20)		N/A
	White		127 (66.8)		55 (43.3)		72 (57.7)		N/A
	Other		14 (7.4)		8 (57.1)		6 (42.9)		N/A
	Patient declined to answer		1 (0.5)		1 (100)		0		N/A
**Ethnicity, n (%)**								.68^d^	
	Non-Hispanic		178 (93.7)		93 (52.2)		85 (47.8)		N/A
	Hispanic		12 (6.3)		7 (58.3)		5 (41.7)		N/A
Use of an interpreter during visit, n (%)		25 (13.2)		19 (76)		6 (24)		.01	
Medicaid beneficiary, n (%)		36 (18.9)		30 (83.3)		6 (16.7)		<.001	
Active patient portal account, n (%)		137 (72.1)		56 (40.9)		81 (59.1)		<.001	
Presence of caregiver during visit, n (%)		48 (25.3)		17 (35.4)		31 (64.6)		.01	
**Specific functional impairments, n (%)**									
	Hearing problems		87 (45.8)		51 (58.6)		36 (41.4)		.13
	Vision problems		123 (64.7)		69 (56.1)		54 (43.9)		.20
	Cognitive problems		65 (34.2)		38 (58.5)		27 (41.5)		.25
**Provider type for visit, n (%)**								.12^e^	
	Behavioral health		17 (8.9)		12 (70.6)		5 (29.4)		N/A
	Geriatric medicine		173 (91.1)		88 (50.9)		85 (49.1)		N/A
	Acute visit		83 (43.7)		40 (48.2)		43 (51.8)		.28
**Vitals obtained during visit, n (%)**									
	Blood pressure		56 (29.5)		26 (46.4)		30 (53.6)		.27
	Heart rate		28 (14.7)		10 (35.7)		18 (64.3)		.05
	Temperature		14 (7.4)		7 (50)		7 (50)		.84
	Oxygen saturation		11 (5.8)		3 (27.3)		8 (72.7)		.08
	Weight		19 (10)		13 (68.4)		6 (31.6)		.15
After visit summary viewed by patient, n (%)		85 (44.7)		20 (23.5)		65 (76.5)		<.001	
Number of medication changes (mean, SD)		0.87 (1.3)		0.8 (1.1)		1.0 (1.5)		.33	

^a^Percentages were calculated using the values in the All Visits column as the denominator.

^b^*P* value is based on a comparison between White patients and non-White patients in terms of whether they had telephone or video visits.

^c^N/A: not applicable.

^d^*P* value is based on a comparison between Hispanic patients and non-Hispanic patients in terms of whether they had telephone or video visits.

^e^*P* value is based on a comparison between behavioral health and geriatric medicine in terms of which provider type was used for telephone or phone visits.

With respect to visit characteristics, almost half (83/190, 43.7%) of the visits were for acute reasons. The majority of appointments (173/190, 91.1%) were with medical clinicians instead of psychologists (17/190, 8.9%). The average duration of visits was 33.6 minutes (SD 10.4 minutes). The most common vital sign reported was blood pressure (56/190, 29.5%), whereas only 7.4% (14/190) and 5.8% (11/190) of appointments recorded temperature and oxygen saturation, respectively.

Of the 190 reviewed visits, 47.4% (n=90) of appointments used video. Of these visits, 56.7% (51/90) used Doximity, an independent HIPAA-secure video platform, 42.2% (38/90) used the video platform in the EHR, which was accessed via an active patient portal account, and 1.1% (1/90) used FaceTime. The physical exam for 71.1% (64/90) of these visits included visual observation-dependent findings. The main reason cited for not using video was a lack of equipment (54/100, 54%). Other common reasons for not using video included patient preference (32%, 32/100) and cognitive problems (23%, 23/100). Of the 100 telephone visits, 56% (n=56) of patients had active patient portals.

With regard to the visit outcomes (Table 2), video visits were an average of 7 minutes longer (mean 37.3 minutes, SD 10 minutes) than telephone visits, after adjusting for age, use of an interpreter, Medicaid coverage, and presence of a caregiver (*P*<.001). On average, video visits documented 20% more visit diagnoses than telephone visits, after adjusting for age, use of interpreter, Medicaid coverage, presence of caregiver (*P*=.001). The rates of advance care planning discussion between video and telephone visits did not significantly differ.

**Table 2:** Telemedicine visit outcomes listed by visit format (N=190).

Variable	All visits	Telephone visit^a^ (n=100)	Video visit^a^ (n=90)	*P* value^b^
Duration of visit, minutes (mean, SD)	33.6 (10.4)	30.3 (9.7)	37.3 (10.0)	<.001
Number of visit diagnoses (mean, SD)	5.1 (2.8)	4.5 (2.5)	5.7 (3.0)	.001
Medical power of attorney discussion, n (%)	31 (16.3)	19 (61.3)	12 (38.7)	.29
Code status discussion, n (%)	32 (16.8)	18 (56.3)	14 (43.7)	.65

^a^Percentages were calculated using the values in the All Visits column as the denominator.

^b^Adjusted for age, use of an interpreter, Medicaid coverage, and presence of a caregiver.

## Discussion

We described the rapid implementation of telemedicine visits that utilized both telephone and video visits to meet the needs of geriatric primary care patients early in the COVID-19 pandemic. Nearly half of the telemedicine visits conducted were video visits, and the 7-minute difference between video visit duration and telephone-only visit duration may represent a clinically meaningful difference. In other contexts, longer visit durations improve patient satisfaction [19], and many additional interventions, such as smoking cessation counseling, can be done with longer visits [20]. The higher number of visit diagnoses during video visits supports the notion that longer visit duration is related to an increased number of problems addressed in an appointment.

Despite the shorter duration, telephone visits had similar rates of advance care planning to those in video visits. This suggests that clinicians are comfortable with having these discussions during either visit type, even without visual cues. In the setting of the COVID-19 pandemic, Medicare has temporarily extended the coverage of advance care planning to audio-only visits [18]. Our findings support this policy change and suggest that this coverage should be continued after the pandemic.

Although most studies on videoconferencing visits have included the home monitoring of vital signs [9,10,12,16], most appointments in our study lacked vital sign monitoring. When vital signs were available, they were frequently limited in number. As such, increasing access to vital sign monitoring at home is necessary to put current telemedicine research into practice.

Although our findings demonstrate that some older adults were able to use video visits, a slight majority of patients were unable to access this visit format, as over half of visits were audio-only. The limited use of videoconferencing compared to telephone visits was also described in recent data from the Veterans Health Administration [21], a multisite geriatric clinic in Michigan [22], and the Centers for Medicare and Medicaid Services [23]. Social factors had the biggest association with the visit format. Racial and ethnic minority patients, those without caregivers present at visits, those requiring interpreters, and those with Medicaid were significantly less likely to have videoconferencing visits. Based on the results of this study, further work and policy changes are needed to ensure that racial and ethnic minority patients and those with fewer resources have access to video visits in order to minimize the risk of further exacerbating health disparities within underserved groups.

Interestingly, 40.9% (56/190) of patients that had telephone visits had active electronic patient portals. This suggests that having access to the equipment required for this portal (ie, a computer or smart phone with internet access) is not sufficient in navigating the videoconference platforms. A lack of training for electronic patient portals is a common barrier for patient use [24]. An alternative explanation is that patients’ family and caregivers, rather than the patients themselves, set up the portal to communicate with patients’ medical teams on their behalf. Since clinician documentation does not routinely describe whether another person assisted patients with video visits, we also did not know the role that family caregivers and other home-based supporters may have had in facilitating the visits.

There are notable limitations in this study. Chart review limited the scope and precision of the data collected and subjected results to errors in documentation. The results of this study may not apply to older adults seen in nongeriatric practices, those who have fewer comorbidities, and those with limited access to clinical resources for supporting telemedicine visits, such as patient portals. Furthermore, our study population was predominantly White and without Medicaid, and therefore may have more access to technology than other populations.

There are calls for increasing the role of telemedicine, even after the pandemic [23,25], but there is still much to learn about telemedicine appointments, including their potential impact on the quality of care and patient satisfaction. Future investigations should focus on addressing disparities in accessing videoconferencing, the quality of virtual and nonvirtual visits measured by patient satisfaction surveys and other methods, and optimal platforms and clinical implementation requirements for virtual visits. Furthermore, given the potential of ongoing reimbursement for telemedicine visits, there will also be opportunities to study the early adopters of older adults with multiple medical conditions who routinely use video visits to better understand patient, caregiver, clinic, health system, and community-level facilitators that may promote the ongoing uptake of video visits. This study provides insights on the use of video and telephone visits for geriatric patients that will be important as we continue to deliver telemedicine care remotely.

## Abbreviations

EHR: electronic health record
ICD-10: International Statistical Classification of Diseases, 10th Revision
REDCap: Research Electronic Data Capture
